# Natural Polymeric Materials: A Solution to Plastic Pollution from the Agro-Food Sector

**DOI:** 10.3390/polym13010158

**Published:** 2021-01-04

**Authors:** Maria Assunta Acquavia, Raffaella Pascale, Giuseppe Martelli, Marcella Bondoni, Giuliana Bianco

**Affiliations:** 1 Dipartimento di Scienze, Università degli Studi della Basilicata, Via dell’Ateneo Lucano, 10-85100 Potenza, Italy; maria.acquavia@unibas.it (M.A.A.); giuseppe.martelli@unibas.it (G.M.); 2 ALMAGISI s.r.l Corso Italia, 27-39100 Bolzano, Italy; m.bondoni@almacabio.com; 3 Gnosis by Lesaffre, Pisticci, 75015 Matera, Italy; raff.pascale@gmail.com

**Keywords:** plastic pollution, bioplastics, biocomposites, fruits and vegetables waste

## Abstract

Conventional petroleum-derived plastics represent a serious problem for global pollution because, when discarded in the environment, are believed to remain for hundreds of years. In order to reduce dependence on fossil resources, bioplastic materials are being proposed as safer alternatives. Bioplastics are bio-based and/or biodegradable materials, typically derived from renewable sources. Food waste as feedstock represents one of the recent applications in the research field of bioplastics production. To date, several food wastes have been used as raw materials for the production of bioplastics, including mostly fruit and vegetable wastes. The conversion of fruit and vegetable wastes into biomaterials could occur through simple or more complex processes. In some cases, biopolymers extracted from raw biomass are directly manufactured; on the other hand, the extracted biopolymers could be reinforced or used as reinforcing agents and/or natural fillers in order to obtain biocomposites. The present review covers available results on the application of methods used in the last 10 years for the design of biomaterials obtained from formulations made up with both fruits and vegetables by-products. Particular attention will be addressed to the waste pre-treatment, to the bioplastic formulation and to its processing, as well as to the mechanical and physical properties of the obtained materials.

## 1. Introduction

Over the last 50 years, the global production of synthetic plastics, which are carbon-based polymers such as polypropylene, polyethylene, polyvinyl chloride, polystyrene, nylon, and polycarbonate, has continuously increased and it is expected to double in the next 20 years. Approximately 360 million tons of plastics were produced in 2018 and 17% of it has been produced in Europe [1]. Conventional plastics play a pivotal role in modern society, since they can be readily manufactured to an expanding range of products used in civil and industrial applications thanks to their light weight, flexibility, and durability [2]. However, the huge amount of plastic waste production is one of the most-faced issues over the world both for environmental problems and human health threat [3].

Traditional plastics are almost completely derived from petrochemicals, meaning that fossil feedstocks are used in their production [4,5]. Around 80% of nonfuel chemicals produced by the petrochemical industry are sold for the manufacturing of plastics, thus contributing to environmental pollution as the extraction of oil and gas, particularly hydraulic fracturing for natural gas, releases an array of toxic substances into the air and water, often in significant volumes [5,6,7,8]. Furthermore, at the end of the 20th century, plastics were found to be persistent pollutants in many environmental niches, since they are largely non-biodegradable; therefore, once they reach the environment in the form of macro or microplastics, contamination and accumulation in food chains through agricultural land, terrestrial, and aquatic food chains and water supply could happen. Plastics spread in the environment could easily leach toxic additives or hazardous substances, for example, phthalates, brominated flame retardants, bisphenol A, formaldehyde, acetaldehyde, 4-nonylphenol, and many volatile organic compounds, making them bioavailable again for direct or indirect human exposure [9,10]. Toxic chemicals that enter the human body through microplastics ingestion can lead to several health impacts, including reduced feeding, blocking of the intestinal tract responsible for starvation and impaired bodily functioning, and translocation to the circulatory system [11].

At the end of their life-cycle, plastics product are disposed of by dumping in landfills, by burning in incinerators, or by littering. In the case of littering, plastic wastes fail to reach landfills or incinerators. This is an improper way of disposing plastics and is identified as the cause of manifold ecological problems. Incineration of plastic wastes significantly reduces the volume of waste requiring disposal. It is believed that the volume reduction brought about by incineration ranges from 80 to 95%. However, their burning releases toxic heavy metals and emits noxious gasses like dioxins and furans [12].

Recycling could be thought of as a good way to reduce plastic pollution, as the cost and the GHG emission can be further reduced by implementing optimization strategies [13], however to date it has been less successful due to difficulties in identification and sorting and the presence of various other materials and additives such as fillers and plasticizers which make the process really expensive [14]. As a result, of all the plastic waste generated, the European average of that collected for recycling is only 30%, with very large differences from country to country [1].

In order to deal with the negative effects of plastic pollution, the European Parliament has developed measures aiming at reducing the quantities of plastic waste, approving a new law banning in EU single-use plastic items such as plates, cutlery, straws, and cotton bud sticks, by 2021. As a consequence, many efforts are being made in the scientific world to found bio-based alternatives which could potentially replace them. This has led to the development of a rich and diversified field of research in bioplastic production [15]. Bioplastic materials are obtained from renewable resources and could be biodegradable and/or compostable. The bioplastic aim is to emulate the life cycle of biomass, which includes conservation of fossil resources, CO_2_ production, and water [16]. Bio-based resources are expected to play a key role in the production of novel and bio-based materials, contributing to a reduction of the negative environmental impacts of fossil-based products and thus addressing the bioeconomy of the future [17]. The molecular complexity of plant and bacterial biomass provides a wealth of natural bio-based polymers as well as monomeric feedstocks for bioplastic production. Currently, most bioplastics are produced from agricultural crop-based feedstocks (carbohydrates and plant materials). These, however, are not yet ideally aligned with sustainable development goals (SDGs), due to their competition for arable land, fresh water, and food production [18]. Among the countless renewable biomass sources, food waste (FW) has also received particular attention for biodegradable materials production. The possibility to overcome the problems associated to FW disposal, through its reuse for the manufacturing of bio-based plastics with a reduced carbon footprint, is considered as an attractive proposition in the field of green chemistry.

Recently, several reviews have been published relating to the production of bioplastics starting from food waste [19,20,21,22]. A discussion on economic viability of FW valorization has been offered by Tsang et al. [19], which also reviewed the technologies commonly employed for the production of polyhydroxyalkanoates. Instead, Maraveas et al. [21] gathered on the general aspects of the synthesis of bio-based polymers from agricultural wastes, as well as on their applications. The main steps in the process of biodegradable films elaboration starting from fruit puree were investigated by Matheus et al. [22], especially lingering on the evaluation of the functional properties of the obtained materials, i.e., the antimicrobial and antioxidant properties.

The search for green formulations that can be suitably manufactured for the production of eco-friendly materials is mandatory in the field of green chemistry and environmental sciences. In this review, we will highlight the developments in the application of the methods used over the past decade for the design of biomaterials. Although several reviews discuss on the same topic, here particular emphasis will be given to those obtained from formulations based on fruit and vegetable by-products. In detail, this review represents a comprehensive basis for scientists to direct their research towards natural sources of biopolymers that could be used appropriately for the production of bio-based materials, including cellulose, starch, pectin, cutin-based materials, and biocomposites. A first part will be dedicated to a complete description of the classification systems of bioplastics and to the methods that are commonly used to define their bio-content and biodegradability, as well as their main physical and mechanical properties. Then, a critical discussion on the latest research results concerning bioplastic production from by-products and waste materials of the fruit and vegetable industries made in the last 10 years will be conducted. A summary table (Table A1), containing all the information relating to the pre-processing of the waste, the definition of the green plastic formulation, and the type of manufacturing, as well as the properties of the final material, will be provided.

## 2. Bioplastics: Definition and Classification

According to the European Bioplastics Organization (EBO), a material could be defined as a bioplastic if it is either bio-based, biodegradable, or features both properties [23]. The term bio-based refers to materials or products that are completely or partly derived from renewable resources (biomass); thus the petrochemical resin typical of common plastics is replaced by vegetable or animal polymers and the compounds like glass or carbon fiber or talc are replaced by natural fibers (wood fibers, hemp, flax, sisal, jute) [15]. For those concerning biodegradability, a material could be defined as biodegradable if it undergoes degradation by biological processes during composting to yield carbon dioxide, water, inorganic compounds, and biomass at a rate consistent with those of other known, compostable materials and leaves no other distinguishable or toxic residue [24].

To date, several classification systems based on different criteria have been proposed to distinguish bioplastic materials, as they can show a wide variation in biodegradability percentage and can be derived from a large number of renewable or non-renewable sources [15,25,26,27].

**Figure 1:**
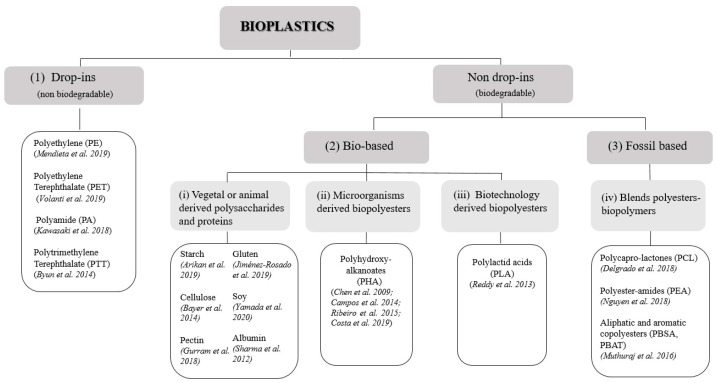
Scheme of bioplastics classification: (1) Drop-in bioplastics (i.e., bio-based or partly bio-based but non-biodegradable plastics), (2) bio-based non drop-in bioplastics, and (3) fossil based non drop-in bioplastics. According to their origin, non-drop-ins (i.e., biodegradable plastics) are divided into (i) vegetal or animal derived polysaccharides and proteins, (ii) polymers from microorganism, (iii) polymers from biotechnology, and (iv) blends of biopolymers and commercial polyesters [25].

Bioplastics could be grouped according to their biodegradability and bio-based content in: bio-based (or partly bio-based) but non-biodegradable plastics (or drop-in bioplastics); biodegradable and bio-based plastics and biodegradable plastics based on fossil sources (Figure 1) [23].

Non-biodegradable bioplastics are obtained from renewable sources and they are comparable to classical plastics for the time needed for their complete environmental degradation. This group of plastics are named “drop-in” bioplastics [28] and nowadays represent one of the largest sectors of the global bioplastics production. Bio-PET (bio-polyethylene terephthalate) represents a very common drop-in bioplastic example. For PET production, an esterification reaction between terephtalic acid (PTA) and ethylene glycol (EG) followed by a polymerization through a polycondensation reaction with water as by-product is used [29]. In the traditional production of PET, both PTA and EG are fossil refinery products: petroleum refiners first separate out *para*-xylene (PX) from BTX (Benzene, Toluene, Xylene) mixtures by crystallization method and then oxidize it to PTA. Similarly, in order to obtain EG, ethylene derived from the alkene co-products of natural gas production are processed through hydration and oxidation [30]. For bio-PET, instead, EG or both monomers are obtained from renewable sources by a process identical to that used for petro-PET and also their technical properties are identical to those of their fossil counterparts [31]. Ethylene glycol is always available on a large scale from biomass: at the beginning cellulose recovered from lignocellulosic biomass is converted into xylitol and sorbitol, which are easily hydrolyzed to EG in the presence of several mono- and bimetallic phosphide catalysts [32,33]. Moreover, bio-ethanol derived from sugar cane or corn stover and glycerol, as a co-product of biodiesel, have been used as a feedstock to produce EG [34]. The production of terephtalic acid by green chemistry processes based on the use of chemical precursors extracted from corn, sugar beet, or orange peel, i.e., isobutanol, 5-hydroxymethylfurfural, and limonene, respectively, is used to a smaller extent [31].

While drop-ins are well known on the market, non-drop-in bioplastics, i.e., plastics that are biodegradable bio-based or based on fossil sources, are alternative materials usually used in niche fields, for example for food services, agriculture, or biomedical applications; therefore, their trade has been emerging only in recent years [28,35,36]. These biodegradable polymers can be classified, according to their origin, into four major categories, namely (i) agro-polymers, (ii) polymers from microorganism, (iii) polymers from biotechnology, and (iv) blend of biopolymer and commercial polyesters [15] (Figure 1).

Starch, cellulose, pectin as well as animal and vegetable proteins, such as casein and gluten, are well known for being feedstock of agro-polymers based bioplastics [37,38,39]. Starch, cellulose, and pectin are polysaccharides that can be extracted from several vegetables and fruits (potato, corn, rice, tapioca, apple) and they are used mainly to produce packaging materials [15]. Often, protein additives are used in order to fabricate materials with novel or improved technological properties. In fact, due to the difference between the elemental composition of proteins (covalent bonds between hundreds of amino acids) and polysaccharides (covalent bonds between monosaccharides with ramifications), their mixtures can evidence a wide variety of two- and three-dimensional structures with different physicochemical and rheological properties [40].

In addition, there are a lot of polymers that could be produced by a range of microorganisms, cultured under different nutrient and environmental conditions [41]. Polyhydroxyalkanoates (PHAs), for example, are linear thermoplastic polymers, with hydroxyalkanoic acid as a monomer unit, which can be synthesized intracellularly as insoluble cytoplasmic inclusions by heterotrophic bacteria, such as *Cupriavidus necator* [42,43], recombinant *Escherichia coli* [44], and also by photoautotrophic microorganisms like microalgae [45]. Their synthesis occurs in the presence of an excess of carbon, when other essential nutrients such as oxygen, phosphorus, or nitrogen are limited; after their extraction from cell cultures, they can be processed in a similar way to that of polypropylene, including extrusion and injection molding, obtaining a material with similar properties as well.

On the other hand, bacterial microorganisms can also be used to produce, in a biotechnological approach, biodegradable polymers through the fermentation of carbohydrates obtained from agricultural by-products such as starchy substances as corn, wheat, or sugar and corn starch. Poly Lactic Acid (PLA)-based bioplastics are obtained from a fermentative process that involves conversion of corn, or other carbohydrate sources into dextrose, followed by fermentation/conversion into lactic acid [25]. Thus, lactic acid is isolated and polymerized to yield a low molecular weight, brittle polymer whose chain length could be increased by using external coupling agents [27].

The last group of biodegradable materials is represented by blends of biopolymers and polymers obtained by chemical synthesis from fossil resources [46,47,48]. Polymers blending is a technique that allows to modify the properties of a material using a conventional technology at low cost. In this way, biodegradable polyesters such as Poly CaproLactone (PCL), which is obtained by the condensation of 6-hydroxycaproic acid or through the ring opening polymerization of ε-caprolactone [49], could be easily used to improve mechanical properties of natural polymers as starch, conferring them a better water resistance due to its hydrophobicity [50].

Nowadays, although with different percentages, all bioplastics are used in a wide range of sectors: from packaging, catering products, consumer electronics, automotive, agriculture/horticulture, and toys to textile fields. The field of application of a given bioplastic material is clearly dictated by its mechanical properties as well as by its bio-based content or its biodegradability. These characteristics are evaluated before bioplastics are promoted on the market.

Moreover, to produce functional materials with biological properties, in the case of bioplastics obtained from fruits and vegetable wastes, a metabolic characterization of the raw material is needed. Among the analytical techniques used to this aim, both chromatography and the mass spectrometry are critical as they allow both targeted and untargeted characterization of the main classes of metabolites occurring in a given matrix [51,52,53,54,55,56,57,58,59,60,61,62].

### 2.1. Bioplastics Bio-Based Content and Biodegradability

Both bio-based and either biodegradable plastic materials have become the world’s most widely choice among bioplastic materials as their production has a low environmental cost compared to traditional plastic ones [63]. To promote the diffusion of either bio-based or biodegradable plastic materials, the Public Procurement Working Group of the European Commission’s Expert Group for Bio-based Products published 15 recommendations in order to enable procurement policies to embrace eco-friendly materials. Due to emerging trade of this type of materials, it is necessary to establish a labelling harmonization, as well as the existence of standards and test methods to define and measure properties and characteristics like bio-based content, biodegradability, and other attributes unique to ready-to-market products.

The bio-based content of a material is the amount of the biomass-derived carbon, as compared to its total organic carbon content (TOC). The carbon content of biobased materials is determined independently and unequivocally as reported in international standard methods of the American Society for Testing and Materials (ASTM) and of the International Organization for Standardization (ISO). In detail, ASTM D6866-20 and ISO 16620-2 methods report radiocarbon analysis as the technique to determine the bio-based content of solid, liquid, and gaseous samples. The employment of the radiocarbon dating method is based on the significative difference in ^14^C isotopic signature between the fossil derived (^14^C-free) and the biomass derived (^14^C-including) materials. In detail, the presence of ^14^C in the bio-based materials is due to the fact that ^14^C containing carbon dioxide formed in the atmosphere, participates in the photosynthetic processes from which the biomass derives. Thus, the ^14^C content of biomass derived materials is the result, in a first approximation, of ^14^C atmospheric levels [64,65]. ^14^C measurements could be done by using Accelerator Mass Spectrometry (AMS) along with Isotope Ratio Mass Spectrometry (IRMS) or by using Liquid Scintillation Counting (LSC) techniques (ASTM International, 2020). In order to define a bioplastic as bio-based, a biomass-derived carbon content not less than 25% is required [66].

**Figure 2:**
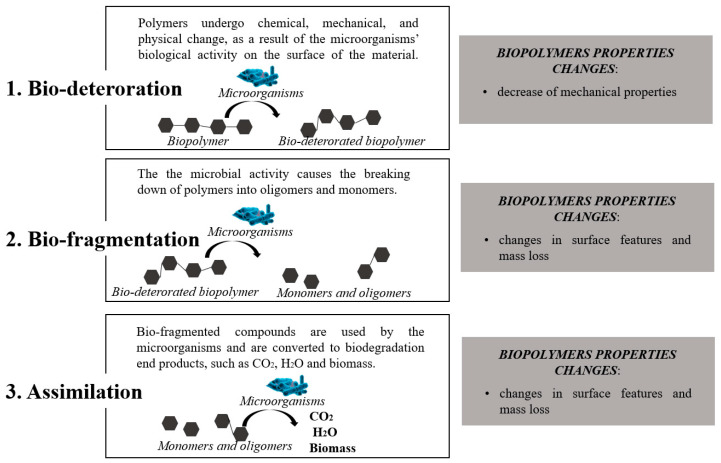
The main three steps through which the biodegradation of polymers occurs: (1) bio-deterioration, (2) bio-fragmentation, and (3) assimilation.

Another key bioplastic property to be measured is the biodegradability [26], which refers to the ability of a material to decompose after interactions with biological elements. The biodegradation of polymers involves three steps: bio-deterioration, bio-fragmentation, and assimilation (Figure 2) [67]. Bio-deterioration is the modification of mechanical, chemical, and physical properties of the polymer due to the growth of microorganisms on or inside the surface of the polymers. In the bio-fragmentation step, microorganisms fragment polymers in oligomers and monomers, which, in the next assimilation step, are available as their carbon, energy, and nutrient sources finally with CO_2_, water, and biomass as by-products [26]. It should be pointed out that only specific microorganisms could degrade a given type of bioplastic. It has been reported that PCL can be degraded by bacteria isolates that exist in deep sea sediments, but these isolates are incapable of degrading other types of bioplastics, such as PLA, PHB, and PBS; however, there exist composting bacteria capable of degrading the latter [26].

The biodegradation of bio-plastic materials is highly dependent on their chemical structures. Generally, polymers with a shorter chain, more amorphous parts, and less complex formula are more susceptible to biodegradation by microorganisms [68]. The presence of additives could influence the biodegradability of a matrix. As an example, polypyrrole, the archetype of polymers integrated in biosensing devices for biomedical applications, can acquire enhanced biodegradability if grafted onto cellulose chains, thus forming biocomposite [69,70]. Moreover, the pH, temperature, and the oxygen content of the environment in which the polymers are placed or disposed of, could be key factors for their biodegradation [71,72]. For example, oxidative-degradable polymers accelerate their decomposition under the effect of oxidation through heat and/or UV light. UV radiation can disrupt polymer chains, since the radiation can be absorbed by oxygen-containing components to initiate a primary degradation; these polymers are known as photodegradable polymers. During photodegradation, both molar mass and crystal structure are affected. The plastics that have the capacity to biodegrade by hydrolytic mechanisms such as biopolymers made of cellulose, starch, and polyesters such as PHA are known ad hydro-biodegradable bioplastics [73].

To date, a wide variety of methods for measuring the biodegradability of polymeric biomaterials have been currently developed and most of them are in agreement with ASTM, ISO, and European Standards (EN) standard methods in terms of environmental conditions, timings, and scales of the tests. Overall, all methods are focused on an indirect measure of degradation process, such as oxygen consumption or biogas generation (CO_2_) by measuring differences of pressure in the test flasks and carbon dioxide production [24]. A biodegradation level higher than 90% in comparison with cellulose (positive standard) in 180 days, under conditions of controlled composting measured through respirometric methods has been established by the European Norm EN 13,432 as the level for a material/product to be defined as biodegradable and compostable. In addition, a disintegration level higher than 90% in three months and the respective ecotoxicity and chemical safety criteria should be kept. Then, only when the products meet the EN 13,432 standard criteria can the wording “biodegradable” be reported on the packaging label.

The biodegradation of bioplastics has beenextensively investigated in soil and compost environments, where they mainly showed high degradability [26]. Anyway, the conditions of the experiments conducted to study the bioplastics biodegradability are highly variable, and to make a clear comparison among them is difficult. The experiments carried out in compost or in anaerobic digestion environments show a biodegradability over 50% in 65 and 68%, respectively. For those carried out in aquatic environments, this share is 44%, and for experiments carried out in soil, it is 33% of the cases [73].

It should be pointed out that, in addition to increasing bio-based content and biodegradability, bioplastics intrinsic properties often need to be improved to meet industrial expectations. The optimization can concern, for example, mechanical properties, increased material flexibility, increased rigidity, increased resilience, and improvement of water absorption capacity [74,75,76].

### 2.2. Bioplastics Mechanical and Physical Properties

In order to assess the suitability of a biomaterial for a given sector and to establish the service life that can be expected, an evaluation of its mechanical-physical properties is mandatory. The main mechanical properties that are typically tested after the production of a bioplastic are the ultimate tensile strength, the Young’s Modulus, and the elongation at break. The ultimate tensile strength, or just tensile strength, indicates the maximum stress that a material can withstand before fracturing, while the Young’s Modulus, also known as elastic modulus, defines the stiffness of a material: the bigger is its value, the stiffer the material [77]. As regard to the elongation at break values, they are a measure of material ductility and depend on the rate (crosshead speed) and the temperature. The elongation at break value is, generally, very small and close to zero for brittle materials. On the contrary, materials with a better capacity to handle an excessive load without failure show higher elongation than 100% [78]. Clearly, all these properties are affected by the chemical structure, the orientation degree of the polymers, and the crystallinity of the material, as well as by the eventual presence of fibers that act as reinforcement, or plasticizers [79,80]. Plasticizers are low volatile molecules, added to bio-polymeric materials to ensure an increasing of their extensibility, dispensability, flexibility, and elasticity [81]. Several theories to explain the mechanisms of plasticization action have been proposed [82]. The lubrication theory states that plasticizers, by interspersing themselves, act as internal lubricants by reducing frictional forces between polymer chains. The gel theory, instead, postulates that the rigidity of polymers comes from three-dimensional structures, and plasticizers take effect by breaking polymer-polymer interactions (e.g., hydrogen bonds and van der Waals or ionic forces). The free volume theory states a plasticization as a study of ways to increase free volume and is useful in explaining the lowering of the glass transition temperature (*T*_g_) by a plasticizer. Ideal plasticizers should be miscible and compatible in all proportions with plastic components, and they may be added to polymers in solution (dispersion technique) or after solvents have been removed (absorption technique) [83,84]. Water, oligosaccharides, polyols, and lipids are different types of plasticizers widely used for edible films and coatings [85].

**Figure 3:**
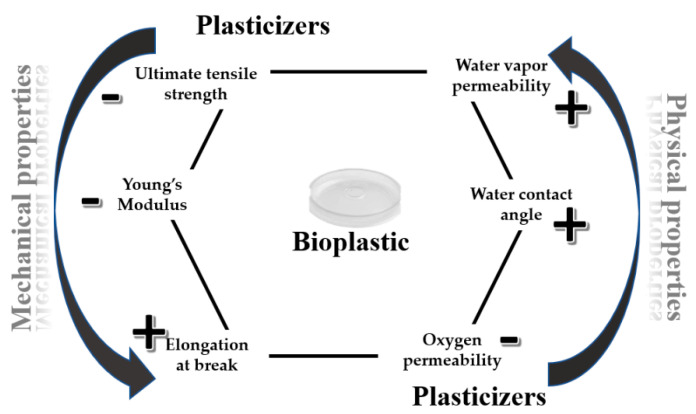
Effects of the plasticizers’ addition on mechanical and physical properties of bioplastic materials.

For hydrophilic polymers, polyols have been proven to be very efficient as plasticizers [86,87]. In detail, for bio-based polymers obtained from fruits and vegetables waste, the recent researchers have focused on the usage of glycerol [88,89,90,91,92] and sorbitol [93]. Glycerol content has significant effects on the mechanical properties as well as on the dynamic rheological behavior of thermo-molded bioplastics. Indeed, it was demonstrated that the increasing of glycerol content decreases tensile strength and Young’s modulus but improves ductility at room temperature [94]. Several studies on plasticization of chitosan films revealed that poly(ethylene glycol) (PEG) could improve the elastic properties of the chitosan biopolymer. Caner et al. [95] observed that chitosan plasticization using PEG was stable until nine weeks of storage (Figure 3).

In addition to the mechanical properties, plasticizers also affect the physical properties of the biomaterials, which means water vapor permeability (WVP), oxygen permeability (OP), and water contact angle (WCA) (Figure 3) [46]. These parameters serve as indicators of how easily water vapor or oxygen can penetrate a biodegradable material and they are a function of the hydrophilicity and hydrophobicity ratio of the main components by which the biomaterial is made. As to water contact angle, which is measured as the angle between the baseline of a drop deposited on the surface of the material and the tangent at the drop boundary, it increases with increasing surface hydrophobicity [87]. Since the surfaces degree of hydrophobicity is important to ensure good barrier properties, the evaluation of WVP, OP, and WCA is demanded. Recently, Aguilar et al. [96] found that different physical and mechanical properties could be achieved at room temperature for bioplastics based on a soy protein isolated as a byproduct of the soy oil industry and added with different polyols, i.e., s (glycerol (GLY), ethylene glycol (EG), diethylene glycol (DEG) and triethylene glycol (TEG). In this sense, TEG-bioplastics were opaque, brittle, and also had a higher water uptake capacity, while EG-bioplastics were more ductile and translucent, absorbing much less water when immersed. Only GLY and TEG remained in the bioplastic after 9 days of storage at 50 °C, pointing out the volatility of EG and DEG causing a major ageing effect. On the other hand, it was also observed that sugars like sucrose and trehalose could act as plasticizers in presence of water. In detail, when water is included in the bioplastic formulation together with glycerol, sugars are solubilized within the aqueous fraction, and then play a plasticizer role in the bioplastics. In that case, lower viscoelastic properties and greater water absorption ability are generally detected [97].

## 3. Food Waste as Feedstock for Bioplastic Production

The most recent research concerning bioplastic production focuses on by-products and waste materials of food industries. According to the Food and Agriculture Organization (FAO) of the United Nations, every year an estimated 1.3 billion tons of food is wasted globally from all stages of the food supply chain including post-production, handling/storage, manufacturing, wholesale/retail, and consumption. Since food waste landfilling yields undesirable results, such as greenhouse gas (GHG) emissions and groundwater contamination, their valorization through bioplastics production could offer the possibility to overcome their disposal problem by renewable sustainable processes [19]. In addition, the production of value-added products while reducing the volume of waste is expected to reduce the production cost of biodegradable plastics, e.g. compared to conventional routes of production using overpriced pure substrates [19].

**Figure 4:**
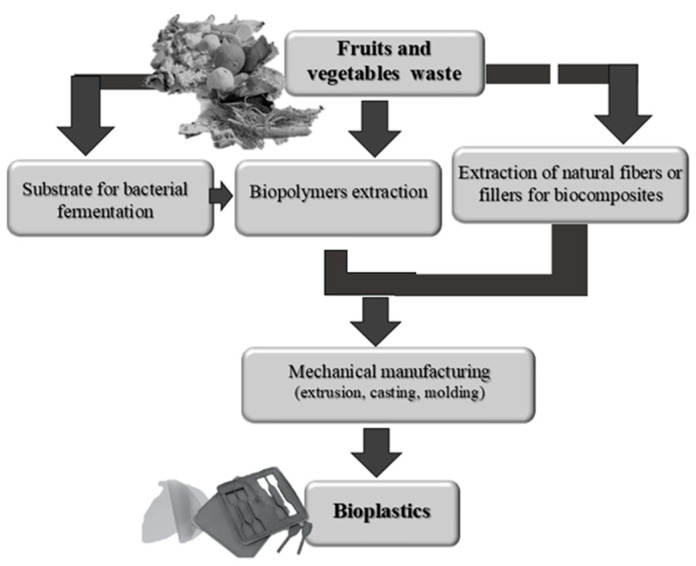
Conversion of food wastes into bioplastics could occur through biopolymers extraction and their mechanical manufacturing (extrusion, casting, molding, or combination of them). In more complex processes, food wastes are used as substrate for bacterial fermentation in order to produce biopolymers or as raw material for the extraction of other natural components, such as fibers, which act as reinforcing agents and/or natural filler of biocomposites.

Food waste (FW) can be valorized in several ways in order to produce bioplastics (Figure 4).

It is often used as substrate for bacterial fermentation to obtain natural polyesters, namely PHA and polylactic acid (PLA). When used to produce PHAs, food waste is a prime candidate for an inexpensive carbon source, due to its widespread availability and the potential to solve significant waste problems. In this case, physical, thermo-chemical, and biological pre-treatments of the FW are requested. Briefly, as reported by Tsang et al. [19], a preliminary liberation of monomers from the FW (e.g., lignocellulosic components) with increasing accessibility of proteins, lipids, and polysaccharides (e.g., starch and cellulose), for subsequent enzymatic hydrolysis and fermentation, are essential. After the pre-treatment, the FW is ready for fermentation step in presence of bacteria, by using several cultivation strategies.

Other simple technologies for the production of bioplastics involve the direct extraction, from the food-waste-stream, of the biopolymeric components that are worked to give the finished products. More complex processes, on the other hand, require additional steps through which the biomass or the biopolymers extracted from it are used as reinforcements or fillers for the realization of biocomposites [98].

In many cases, the different “bioplastic formulations” need to be blended with additives in order to optimize some properties of the materials, such as thermal instability, high water vapor, brittleness, and low melt strength. Plasticizers, like glycerol, for example, are often required to improve the processability and the mechanical properties by interrupting hydrogen bonding and reducing the interactions between the biopolymers chains [81].

However, both formulations, biopolymerics and biocomposites, are lastly processed to obtain biofilms or three-dimensional objects by applying conventional mechanical techniques: extrusion, molding, casting, or a combination of them [88,99,100]. All these processing methods selected for the manufacture of food waste-based bioplastics play an important role in their final properties [101]. Extrusion is a highly efficient way for the continuous shaping of biomaterials, and it consists in pushing the bioplastic dough against an orifice with the desired geometry and dimensions. The mass of the dough inside the extrusion chamber is compacted, and the semi-finished product that comes out is cut to give the wanted length. Instead, with casting and molding, the dough is respectively poured or pressed against a rigid frame.

Compression molding technique has been widely employed for the development of biofilms or 3D objects without the use of any solvent or binder [88,102]. With this technique, the waste of interest or its dried extract is finely powdered and subjected to high temperatures and pressures through a heated press. Under this thermo-mechanical treatment, proteins undergo denaturation and dissociation leading to the formation of new links and their aggregation to new forms; in addition, biopolymers show a self-binding ability that is exploited to produce three-dimensional objects. Furthermore, the molding method is more suitable for industrial applications since it is characterized by lower energy demand and processing time compared to other techniques like solution casting [102].

In the last 10 years, several food wastes have been used as raw material for the production of bioplastics, including biocomposites; mostly fruit and vegetable wastes rich in polysaccharides (such as cellulose, starch, pectin) and in fibers [90,93,101,103,104,105] (Table A1). In the next paragraphs, a focus on the main uses of fruits and vegetables wastes for the production of bioplastics will be provided.

### 3.1. Biopolymers-Based Plastics

Biopolymers extracted from fruits and vegetables wastes show different characteristics and properties that make them more or less suitable for the production of eco-friendly materials (Table 1). The extraction of biopolymers from food waste could be achieved chemically or enzymatically. Enzymatic processes are widely considered “clean” since they are solvent-free [106]; however, this technology is still hindered by economic and technical limits, i.e., costly enzymes and long processing period. Because of the high cost and time-consuming nature, the production of bioplastics through sugar’s bacterial fermentation, occurring in agricultural waste, is disadvantageous. Therefore, chemical extraction with solvents could be considered as the best solution at the lowest amount of energy.

One of the main macromolecules extracted from fruits and vegetables waste and used for the production of biomaterials is the cellulose. The preparation of the pure cellulose bioplastics from bio-sources is not easy due to the highly structured intermolecular hydrogen bonding network of the polymer, which cannot be melted or dissolved by standard processes such as thermoforming [37,107]. Thus, the cellulose is usually used in industrial applications in the form of derivatives, such as esters or ethers, from which cellulose is then regenerated [108]. Nevertheless, in recent years, several biomaterials have been manufactured through amorphous cellulose extracted from vegetables by using different solvents. Bayer et al. [37] obtained amorphous cellulose-based biomaterial by digesting parsley and spinach stems, rice hulls, and cocoa pod husks wastes in trifluroacetic acid (TFA), followed by casting and evaporation. TFA is a naturally occurring and biodegradable organic acid that can co-solubilize cellulose with other contained organic matter; it breaks the hydrogen bonds between neighboring cellulose chains (intersheet hydrogen bonds) and partial trifluoroacetylates OH groups of cellulose with formation of amorphous materials [109]. The mechanical properties of the produced cellulose-based biofilms were proved to be largely dependent on the starting biowaste. Indeed, cocoa pod husks biofilm displayed a tensile stress at break of approximately 30 MPa; whereas for the rice, the parsley, and the spinach-derived films, the obtained values were, respectively, 7 MPa, 5 MPa, and approximately 1 MPa, i.e., values close to elastomers and low density polyethylene thermoplastic [37]. Such higher stresses at break and strains for cocoa pod husks derived biomaterial were due to their significant number of triglycerides, i.e.oligomeric esters precursors of biopolymers. Instead, residual silica in the rice hulls derived material conferred a higher rigidity compared to parsley- and spinach-based biomaterials. UTS (ultimate tensile strength) at high Young’s modulus comparable to poly(ethylene terephthalate) of bioplastics from cocoa pod husk could be compared with petroleum-based thermoplastics, such as high-density polyethylene and polypropylene. Rice straw was used also by Bilo et al. [110] to produce a new cellulose–based bioplastic material through a process that involved the digestion with TFA, preceded by an extraction pre-treatment performed in a rapid dynamic solid-liquid extractor. With this process, a bio-material with better mechanical properties, compared to those evaluated by Bayer et al. [37], was obtained. Indeed, the tensile test of dried and wet dumbbell specimens allowed to ascertain tensile strengths and elongations at break equal to 45 MPa and 6.1% and 10 MPa and 63%, respectively (Table A1). The replacement of TFA with a diluted aqueous cloridric acid (HCl) solution has been found to be a better method to obtain biofilm evidencing higher stiffness and lower ductility. Perotto et al. [111] used this water-based process to convert carrot, parsley, radicchio, and cauliflower wastes into flexible bioplastic films made by cellulose crystals fused together, with some soluble components like pectin and sugars blended homogeneously acting as plasticizers. Compared to the oil-based polymers, the Young’s Modulus (1.3 ± 0.2 GPa) and the UTS (38 ± 5 GPa) of the carrot bioplastics are similar to those of polypropylene, albeit with lower elongation. The mild conditions of the conversion process were demonstrated also to preserve the functional properties of the original vegetable, like the anti-oxidant activity [111]. An aqueous HCl solution was used also by Yaradoddi et al. [89] to produce a cellulose-based biofilm from banana peels; anyway, in this case, no mechanical tests were conducted in order to evaluate the strength of the material.

Although TFA and HCl are efficient acids for vegetable waste derived-cellulose dissolution, their utilization, and waste production remain problematic if considering the principles of green chemistry. Recently, a less harmful acid, i.e., citric acid, has been used by Liu et al. [112] in a green, non-toxic, waste-free method of synthesizing hydrophobic bioplastic films from spent tea leaves. The resultant material exhibited an ultimate tensile strength of 6.16 MPa and an elongation at break of 13.33%, thus it had a lower stiffness compared to oil-derived polymers, such as PP (Table A1). Since citric acid was found to not fully react with the tea waste matrix, the authors hypothesized that unreacted citric acid acted as a hygroscopic plasticizer in the bioplastic films.

In addition to cellulose, starch, i.e., a polymer consisting of a long chain of two glucose units joined together, namely branched polymerized amylopectin and amylose, can be considered as an effective eco-solution for the production of biomaterials, because it is inexpensive and easily available (Table 1). Starch for the production of biofilm have been obtain from different sources, principally potatoes, banana and cassava peels [90,113,114,115,116,117,118]. Arikan et al. [114] investigated the production of bioplastics from potato peels waste, obtaining satisfactory results in terms of biodegradability (the time requested for the complete biodegradation of the material was 28 days, see Table A1). However, native starch-based films are limited to high water affinity and brittleness, therefore other natural biopolymers are often added as fillers to modify and improve films’ properties. As example, Dasumiati et al. [116] and Fathanah et al. [117] improved the mechanical properties of cassava peels derived starch by introducing chitosan as filler. In another work, proteins derived from soybeans waste were mixed with starch and glycerol as plasticizer, since proteins structure consists of stable three-dimensional networks which do not ensure material with enough plasticity [119]. Instead, Sultan et al. [90] developed bioplastic film from a combination of banana peels derived starch and different concentrations of corn starch (1% up to 5%) as co-biopolymer. Based on the results obtained, the film with 4% of corn starch gave the highest tensile strength 34.72 N/m^2^ compared to other samples, while the authors stated that the biofilms with 3% of corn starch were resistant to water uptake by absorbing water up to 60.65% (Table A1). However, it should be considered that this value is considerably higher compared to conventional plastics such as PP, whose percentage of water absorption after 24 h of immersion ranges between 0.01 and 0.03.

Besides being a starch source, banana peels have been shown also to contain a good percentage of pectins [120]. Pectins are a family of covalently linked galacturonic acid-rich plant cell wall polysaccharides with functions in plant growth, morphology, and development; they also serves as gelling and stabilizing polymers in diverse foods [121]. The production of pectin-based biofilms typically involves the introduction of cellulose and hemicellulose components, since polysaccharidic films show poor tensile and barrier properties compared to those of petroleum-derived polymers. To this regard, Oliveira et al. [75] isolated pectin from banana peels in order to prepare a biofilm whose tensile strength was increased through the addition of cellulose nanocrystals (CNCs) extracted from the same banana wastes (tensile strength values obtained were about 7 MPa, see Table A1). The tensile strength increase was due to favorable nanocrystal–pectin interactions as well as to the reinforcing effect through stress transfer at the nanocrystal–pectin interface [122].

Aside from the poor mechanical properties, the strong hydrophilic character of polysaccharidic films makes them dissolve in contact with water, limiting their applications [75]. To overcome the high water permeability, citric acid could be added, as it crosslinks polysaccharide films by forming covalent diester linkages between two of their carboxyl groups and hydroxyl groups of different polysaccharide chains [123]. In the previous mentioned work reported by Oliveira et al. [75], the presence of citric acid was ascertained to decrease the water vapor permeability from 3.31 to 3.10 g·mm·kPa^−1^·h^−1^·m^−2^. Citric acid was used also for the processing of orange and apple wastes (OW and AW) in order to obtain a biodegradable material through a casting method in which cellulose and hemicelluloses were suspended in the pectin solution and further dried to a film [88,92]. In detail, Batori et al. [92] used a solution of citric acid and glycerol to form a biofilm from OW, exploiting the gelling ability of pectin and the strength of its cellulosic fibers. The tensile strengths of the films were 31.67 ± 4.21 and 34.76 ± 2.64 MPa, respectively, for the oven-dried and incubator-dried films. These values were within the range of different commodity plastics. In addition, anaerobic digestion was performed for testing the biodegradability of the material and a time of 15 day was requested to reach 90% of degradation. Instead, from a mixture of apple pomace waste (AW) and glycerol, a fluffier and connected structure (tensile strength 3.27 ± 0.31 MPa without including a washing step) was obtained by Gustaffson et al. [88], but with significant flexibility, similar to those of PP (elongation %: 55.41 ± 5.38, Table A1).

The same authors [88] made an attempt to produce bioplastics by using solvent-free mechanical processing of AW. Compression molding technique has been widely employed for the development of pectin-based biofilm or 3D objects without the use of any solvent or binder [88,102]. Gurram et al. [102] applied a compression molding method for production of bioplastic films from citrus peel derived pectin. Moreover, free sugars and water-soluble nutrients were extracted from citrus waste and employed for cultivation of the filamentous fungus Rhizopus oryzae, whose biomass was incorporated into the pectin films. The addition of fungal biomass (up to 20%) enhanced the tensile strength (16.1–19.3 MPa) and reduced the water vapor permeability of the pectin films (Table A1).

In addition to the cellulose, starch, and pectine, a sustainable melt polycondensation of unsaturated and polyhydroxylated fatty acids recovered from tomato pomace agro-wastes, has been recently carried out in order to obtain an aliphatic polyester type of bioplastic without the use of solvents during the reaction [124]. Polyhydroxylated fatty acids are found in tomato pomace in the form of cutin, i.e., a biopolyester mainly composed of C_16_ and C_18_ fatty acids monomers linked together and forming an amorphous and flexible three-dimensional polymer matrix [125]. Since cutin isolation to produce bioplastics is a long multistep process and unsuitable for large-scale applications, a direct depolymerized of tomato pomace through alkaline hydrolysis, followed by monomers polycondensation, has been proposed by Heredia-Guerrero et al. [124] as a simpler and cheaper alternative. To that purpose, the influence of different temperatures, reaction times, and amounts of tin (II) 2-ethylhexanoate used as a catalyst, was evaluated. Synthesized tomato pomace bioplastics showed an amorphous molecular structure, whose mechanical properties were dependent on the degree of polymerization. In detail, an increase in hardness of the polyesters synthesized at higher reaction temperatures and amount of catalyst was detected (~1.8 MPa for biopolymers obtained at 125 °C and 0 mmol of catalyst against ~26.3 MPa for biopolymers obtained at 175 °C and 0.1 mmol of catalyst), since in those conditions a higher degree of polymerization was achieved. The water-contact angles of more polymerized samples were around 109°, which are values comparable to traditional hydrophobic polymers such as PDMS and PTFE. Concerning water uptakes, the obtained percentages were typical of low-absorbing plastics (2.1–61%).

### 3.2. Fruits and Vegetables Waste Usage for Biocomposites Production

More often, biopolymers extracted from fruits and vegetable wastes are blended with other polymers whose mechanical and physical properties are not suitable to accomplish commercially acceptable products [100], thus realizing composite materials known as biocomposites.

Biocomposite materials are usually made by a polymeric matrix coming from a renewable and available origin, such as polysaccharides, reinforced by natural fillers. Examples of natural fillers are layered silicates. They can be synthetized from silica naturally occurring in leaves, husks, blades, hulls, roots, and stems of many terrestrial and marine plants, including wheat, rice, horsetails, oats, barley, grasses, and algae. Among bio-wastes, one of the most silica-rich sources is rice husks, which is largely available, typically 20–22 wt% of rice grains. It is been used by Deng et al. [126] for layered silicates synthesis. Layered silicates (LSs) have hydrophilic characteristics owing to the presence of inorganic cations (Na^+^ and Ca^2+^) in the interlayer spacing; hence, they are miscible with different hydrophilic polymers, including starch and pectin, able to compensate their rheological property differences [100].

Despite not being recovered from vegetables wastes, Cokaygil et al. [100] used LSs as natural filler to prepare biocomposite films having corn starch and pectin extracted from orange peels as a polymeric matrix. Different pectin jelly-to-starch weight ratios (63/37, 60/40, 57/43, and 54/46 *w/w*) were considered when formulating the film ingredients. Furthermore, to enhance the compatibility and wettability among starch, LS, and pectin, starch and LSs were chemically modified through reaction with propylene oxide and hexadecyltrimethylammonium chloride, respectively. Among all the films considered, pectin jelly/modified starch-based biocomposite film (54/46 *w/w*) containing 0.25 wt % of LSs was found to be the most promising in terms of texture structure and mechanical integrity.

In the most recent years, wastes of agro-food industries have attracted attention also as sources of natural fibers exploitable as reinforcing elements of biodegradable biocomposite materials. Bio fibers, which are natural polymers, could be obtained from a large variety of fruits and vegetables [127], thus reflecting several characteristic properties unlike conventional fibers. Undoubtedly, conventional fibers for instance glass, carbon, and aramid can be produced with a definite range of properties, with a higher cost as well. In 2013, Schettini et al. [105] developed a novel biocomposite by using hemp and tomato peels and seeds fibers as natural reinforcement for sodium alginate polymer, in order to produce biodegradable pots in agriculture. Three different compositions of biocomposites were prepared by varying the percentage of tomato and hemp fibers added to sodium alginate water solution. By soaking the doughs with a calcium chloride solution, a three-dimensional and stable crosslinked network of calcium alginate was obtained as well and it was subjected to investigation of its functionality, physico-chemical and mechanical behavior. As reported by the authors, by increasing the hemp fibers content, a general enhancement of the mechanical parameters of both un-crosslinked and crosslinked samples was registered, since fibers from hemp strands are more rigid, stiff, and long in comparison to the more flexible and short fibers from tomato peels and seeds [128]. Moreover, crosslinked biocomposites showed a lower rigidity and strength with respect to their corresponding un-crosslinked counterparts (Young Modulus for un-crosslinked 100% tomato fibers biocomposite was 63.62 MPa; while for crosslinked 100% tomato fibers biocomposite Young Modulus was 48.05 MPa). Such a behavior was due to the loss of adhesive properties, which occurs when carboxylated and hydroxyl groups of alginate are strongly engaged in physical interaction with calcium ions during the crosslinking process, thus reducing the bonding strength between the matrix and the fibers [129]. However, these obtained values were all comparable to those of conventional plastics. Instead, Mathivanan et al. [130] used different percentages of pineapple leaf fibers to reinforce tapioca based bioplastic resin through a method based on extrusion followed by hot compression molding. The 30% composition showed the best average modulus value among other composition, leading to the conclusion that the increase of pineapple leaf fibers increases the modulus strength of the composite.

Since passion fruit waste contains about 60% of fibers [131] that, when dried, could be used as reinforcement of thermoplastic starch, Moro et al. [132] tried to develop an extruded starchy bioplastic, reinforced with different content of passion fruit peel (0, 4, 10, 16, and 20%), glycerol and starch mix, recovered from corn and cassava. In this way, it was possible to obtain starch-based bioplastic with stronger and midterm elastic property (Tensile strength ranged between 1.6 MPa and 9.0 MPa, while the elongation at break values were between 24.7% and 54.5%, see Table A1). Despite this, the tensile strength values were lower of oil-derived polymers.

On the other hand, bio-blend of poly-butylene succinate (PBS) and poly-butylene-adipate-co-terephthalate (PBAT) has been recently proved to be strengthened in terms of higher modulus (3.0 GPa) and lower water absorption (3.4%) with the addition of Miscanthus fiber and oat hull followed by reactive extrusion of the dough [133]. Indeed, PBS alone has a tensile strength of around 26.5 MPa, elongation of 21.5%, and a modulus of ~48 MPa. The incorporation of fiber or cellulose remarkably improves the Young’s modulus of neat PBS.

For their rich content in lipids, lignin, and fibrous polysaccharide components (cellulose, hemicellulose), peanut hulls and cocoa shell waste (CW) and hazelnut skin (HS) extracts have also been introduced into synthetic elastomers matrices as reinforcement fillers and plasticizers [76,134,135]. Battegazzore et al. [76] made selective and serial extractions from CW and HS to recover bio-components for producing high-added value PLA and PP plastics. Briefly, a first extraction with diethyl ether mainly separated lipids, phospholipids, and triglycerides, which were worked as plasticizers. In the second extracted fractions, instead, phenolic compounds and flavonoids, such as gallic acid and catechin, were distinguished by UV spectroscopy; therefore, those fractions served as antioxidant and photo-stabilizer for PP. In addition, they positively influenced the PP thermal stability in air; indeed, the temperature of its maximum weight loss was increased from 319 °C to 330 °C and 345 °C by adding HS and CW extracts, respectively. Finally, the last fractions extracted acted as reinforcement filler for PLA and PP; their content linearly influenced the oxygen permeability of the obtained biomaterials (Table A1). Instead, Tran et al. [135] introduced cocoa shell waste powder within an acetoxy-poly(dimethylsiloxane) silicone network through a process that involved a physical mixing with a nontoxic solvent and casting into a mold, with the advantage of direct utilization of CW without any extraction or purification steps. In this case, the antioxidant activity of the final cross-linked bioelastomers was investigated, demonstrating very effective radical scavenging activity against 2,2-diphenyl-1-picrylhydrazyl free radical and 2,2′-azinobis(3-ethylbenzothiazoline-6-sulfonic acid) radical cation.

As PLA reinforcement, cellulose extracted from pumpkins peels, and subsequently acetylated, has also been used [136]. In this case, the addition of 10% of acetylated cellulose enhanced the PLA’s mechanical properties with an increase of the storage modulus at 40 °C of around 40%. More generally, cellulose or cellulose nanocrystals have been obtained from various vegetable or fruit waste, such as banana peels, pine flowers waste, rice straw, palm empty fruit bunch, sago waste, mangosteen peels, and also successfully employed as reinforcements of biopolymers, mainly starch [103,137,138,139,140,141]. In addition, banana pseudostems waste has been recently used to isolate nanocellulose employed for the production of green composites enriched with nano-fillers, such as graphene oxide and nanoclay, and glycerol as plasticizer [91]. As regard to rice straw, besides being considered as reinforcement, its fibers were proved to act as flame-retardant fillers in combination with PLA and lignin by Dahy et al. [142]. Typically, flame retardant used to reduce combustibility of the polymers, are halogen-based additives that act in the vapor phase by a radical mechanism to interrupt the exothermic processes, interfering with the combustion process during heating, pyrolysis, ignition, or flame spread. Instead, a more environmentally friendly alternative that contemplates the incorporation of natural fillers, like rice straw derived fibers, mainly acts to dilute the polymer and reduce the concentration of decomposition gases [143].

## 4. Environmental Impacts of Agro-Food Waste Based Bioplastics Production

Nowadays, fruit and vegetable valorization is one of the main pillars of the circular economy; their use to substitute fossil resources for the production of plastics, is a widely accepted strategy towards sustainable development. In fact, the displacing of conventional plastics with food waste-based bioplastics can lead to considerable energy and GHGs emissions savings [19]. However, it should be noted that this is not always true. Further details about advantages and drawbacks related to the bioplastics use and production are given in Table 2. Despite being promoted as a safer alternative to their oil-based counterparts, bioplastics production involves major drawbacks. Indeed, bioplastics are generally not cost-competitive compared to conventional plastics and their production is plagued by low yields and being expensive. Moreover, some bioplastics have a shorter lifetime than oil-based plastics due to weaker mechanical and physical properties, such as greater water vapor permeability than standard plastic, being easy to tear like tissue paper, or being very brittle. Being compostable and biodegradable sounds great, but many bioplastics must follow a specific disposal procedure and require industrial composting in order to avoid being incinerated or going to landfill. On the other hand, biodegradable polymers require a controlled fate to kickstart the expected biodegradation process and as a result, it is nearly impossible to control and ensure the complete degradation of even potentially degradable plastic materials. Subsequently, when they are disposed of in an uncontrolled fashion, they will accumulate in the environment and fragment into microplastics (MPs). These MPs have proven to display diverse impacts over ingested organisms and ecosystem similar to those of conventional MPs, thus bioplastics could be a solution to MPs only if properly disposed of [144].

As a matter of fact, the employment of fruits and vegetables waste as reinforcement of non-biodegradable polymers in drop-ins significantly increases the energy demand and CO_2_ emission compared to biodegradable bioplastics [145].

Therefore, when the aim is the production of new bioplastic materials from agro-food waste, the effective sustainability of the process should be evaluated. The sustainability of bio-based plastics production depends on several factors that are often summarized in the life cycle assessments (LCAs) of the products [146]. Among them, there are availability of commercially viable quantities of renewable feedstock and agricultural waste, scalable and green production routes, cost and competition with synthetic polymers, and useful life and biodegradation/end of life treatment [21]. Many of these aspects are very often not sufficiently deepened, thus making it difficult to assess environmental impacts associated with the agro-food waste-based bioplastics production.

The greenhouse gas emissions generated by food waste globally represent the third largest emitter in the world, thus any measure to reduce food waste, even to a small extent, may have a significant impact on overall environmental footprint [147]. However, even though the number of fruits and vegetable wasted every year are estimated to be around 484 million, the volume of waste produced does not predict the availability of agricultural waste for conversion into biomaterials. Indeed, a large quantity is employed in other competing applications such as bio-fertilizer and biogas production [148]. In addition, not all the routes proposed for obtaining bio-materials are applicable on a large scale, since sometimes they require extensive and advanced processing. This mainly concerns biocomposites production, which is often based on obtaining fillers and reinforcements, such as cellulose nanocrystals, through complex treatment of the agro-waste [103,122]. On the contrary, more feasible and scalable processes allow bioplastics production after chemical extraction of agro-polymers from the food waste stream. A low environmental impact is associated with this step, as no harsh chemicals, like pyridine and diethyl ether, are used for the production of PHA, and potential occupational hazards are covered.

Regarding the end of life of the agro-food waste = based bioplastics, reuse and recycling are preferred solutions to energy recovery or disposal. However, to date, for materials other than bio-PE or bio-PET, there is no recycling stream established yet [23]. An alternative is their composting, i.e., their aerobic biodegradation under controlled conditions of temperature, humidity, and aeration [72]. Compostability is a clear benefit of agro waste-based bioplastics compared to conventional plastics, resulting in the creation of more valuable compost.

## 5. Bioplastics Market

Currently, the number of bioplastics produced annually in all the world represents only about one percent of the 360 million tons of plastic materials produced globally. However, due to the growing sensitivity towards the adoption of a “green and circular economy” dependent policy, the global bioplastics production capacity is set to increase from around 2.11 million tons in 2019 to approximately 2.43 million tons in 2024 [23].

With a view to regional capacity development, Asia remains a major production hub with over 50 percent of bioplastics currently being produced there. Presently, only one-fifth of the production capacity is located in Europe. This share is predicted to grow to up to 27 percent by 2023. The expected growth will be supported by recently adopted policies in several European Member States, such as Italy and France.

Innovative biopolymers, such as bio-based PP, bio-based PET, bio-based PA, and PHAs continue to drive the growth in bioplastic production. To date, they make up for 40 percent (0.8 million tons) of the global bioplastics production capacities. Bioplastics materials are currently used in an increasing number of markets: From packaging, catering products, consumer electronics, automotive, agriculture/horticulture, and toys to textiles. Among these several market segments, electronics is the less developed (only about 2% of the global bioplastic production concerns this segment), while packaging remains the largest field of application for bioplastics since around 54% of the global bioplastic production is used to serve the packaging industry, including shopping bags producers, plastic bottles producers, and food packaging industry [23].

Biodegradable shopping bags are made of polymers that degrade, or decompose, when exposed to air, water, or sunlight. There are three main types of biodegradable bags, i.e., (1) biodegradable bags made from resins containing starches, polyethylene, and heavy metals such as cadmium, lead, and beryllium, (2) biodegradable bags made by using starches combined with biodegradable polymers such as PLA, and (3) oxo-biodegradable bags, which use Totally Degradable Plastics Additives (TDPAt) to stimulate the breakdown of polymers and thus speed up the biodegradation process of conventional plastics.

As regard to food packaging, in the USA premarketing approval by the Food and Drug Administration is required to ensure that materials are wholesome, safe, and effective [149]. On the other hand, in Europe, food contact materials regulations sets specific manufacturing goals to assure a good quality control system and specifies the thresholds according to the form and composition of polymers, which shall explicitly be authorized in order to preserve food safety (European Commission, 2006; European Commission 2011). Anyway, biobased materials are mostly used to pack short shelf-life products or long shelf-life ones, which do not need very high oxygen and/or water barrier properties, such as fresh fruits, vegetables, pasta, and chips [150]. Actually, biomaterials available show such a wide range of properties, that they are also applicable as packaging materials for other food products, which request stricter conditions, like Modified Atmosphere Packaging (MAP).

Bioplastic materials also offer several advantages in the agriculture sector. Eight percent of the global production of bioplastics is covered by the agriculture and horticulture segment. Examples of bio-based products used in agriculture are mulching films and pots [105,151]. Soil mulching is a practice used in cultivation, which allows weed suppression, reduces the loss of moisture from the soil, and may promote the increasing of soil temperature. Ploughing-in of bio-based and biodegradable mulching films after use instead of collecting them from the field and cleaning off the soil is a more practical and time saving solution. In the same way, bio-based pots are used.

For the automotive field, instead, components made completely or partially from bioplastics can provide a safety standard, that is of ultimate importance in the transportation sector. The products include seat and airbag covers as well as steering wheels. Some of the bio-based plastics such as bio-based polyamides and bio-based polyesters are already successfully being used by leading automotive brands around the world today with the aim of reducing their products’ environmental impact. For example, Toyota typically uses bio-based polypropylene/polylactic acid (PP/PLA) composite derived from plant materials for the realization of up to 60% of the interior design of cars.

Biopolymers find applications in several housewares, such as kitchen tools and utensils, washable storage containers and cups, bathroom accessories, toys, hangers, and hooks. For example, hangers from United Colors of Benetton are made of biodegradable polymers. Nontoxic biodegradable polymers are also being used as sutures by surgeons in life-saving heart operations and other procedures. Easily sterilized, the sutures remain strong and intact until the surrounding tissues have healed. The sutures dissolve and are readily metabolized in the body leaving no trace. Moreover, there has been a surge of bioplastic products that are being introduced in the fast-moving consumer electronics sector, such as touch screen computer casings, loudspeakers, keyboard elements, mobile casings, vacuum cleaners, and a mouse for a laptop. SUPLA produced the first bioplastic touch screen computer by using PLA, in collaboration with a Taiwanese company (Kuender).

To date, a lot of companies have been identified as key players in the production of bioplastics and their distribution witnesses that the majority of them are located in Europe. Many of these companies produce sustainable bioplastics made from plant-based renewable resources, like corn, potatoes, and wheat. The land used to grow the renewable feedstock for the production of bioplastics is estimated to be 0.7 million hectares in 2021 and continues to account for 0.015 percent of the global agricultural area of 4.7 billion hectares. Despite the market growth predicted in the next five years, the land use share for bioplastics will only slightly increase to 0.02 percent.

Novamont SpA (www.novamont.com) is one of the major starch bioplastics producers. The trade name of their starch-based bioplastic is “Mater-Bi” and it is provided for a wide range of manufacturers, which use it to make bags, mulching film, disposable tableware, and packaging. Furthermore, Amynova Polymers GmbH (www.amynova.com) is engaged in the production of a starch-based substance named “CropCover”. CropCover is an innovative “adhesive” non-toxic, non-combustible, and fully biodegradable applied together with pesticides and foliar fertilizers, in order to reduce their rinsing during heavy rainfall and to guarantee a longer stay time on the plant. Biotec Biologische Naturverpackungen GmbH & Co. KG (www.biotec.de) and Cardia Bioplastics (www.cardiabioplastics.com) produce and sell a new generation of customized thermoplastic materials too, with various functional properties fully biodegradable and compostable according to EN 13432. Moreover, there are companies that exploit waste as feedstock for bioplastics production; an example is NaturePlast (www.natureplast.eu). Since 2015, NaturePlast has been producing and marketing a range of biocomposites consisting of by-products and plant fibers (such as hemp), sourced mostly from the French territory. The objective is to incorporate by-products or local waste materials in different polymers to ensure a circular economy and the reclamation of waste materials.

## 6. Conclusions

The valorization of food waste (FW) can create opportunities to produce new valuable bioplastics, which represent an eco-friendly alternative to conventional petroleum-based plastics. Bioplastics produced from fruits and vegetables waste are compatible with the “circular economy”, therefore with “zero waste” or more precisely aiming at a complete use of it; moreover, they could create positive synergies between industry and the agro-food sector, with considerable advantages for environmental pollution. This review highlights that the real challenge is to create new eco-friendly materials from food waste and not from specially grown crops, whose production comes at an environmental cost. As the FW potential as raw material for bioplastics production is well known, such a novel perspective focusing on the overall methods used for the design of biomaterials starting from both fruits and vegetables wastes, provided in this review, should be particularly helpful in the fields of the green chemistry and of the environmental sciences.

## Figures and Tables

**Table 1:** Properties associated with the main biopolymers extracted from fruits and vegetable wastes for bioplastics production.

Biopolymer Name	Biopolymer Type	Properties	Fruits and Vegetable Wastes Used as Biopolymer Source
*Cellulose*	Polysaccharide	Highly structured intermolecular hydrogen bonding network; impossibility of melting or dissolution by standard processes such as thermoforming.	Banana peels, carrots waste, cauliflower waste, cocoa pod husks, orange peels, parsley steams, radicchio waste, rice hulls, spinach steams, tea leaves waste.
*Starch*	Polysaccharide	Strong inter- and intra-molecular hydrogen bonding; water sensitivity and poor fowability; brittleness.	Banana peels, cassava peels, potato peels.
*Pectin*	Polysaccharide	Gelling ability but poor tensile and barrier properties; water sensitivity.	Apple pomace, banana peels, citrus waste, orange peels
*Cutin*	Polyester of hydroxy fatty acids	Amorphous and flexible three-dimensional polymer; hydrophobic, low water sensitivity.	Tomato waste

**Table 2:** Main advantages and drawbacks related to the production and the use of bioplastic materials.

	Advantages	Drawbacks
**Production**	Reduction of greenhouse gas emission; saving fossil fuels, possibility of using a local resource, less energy during the manufacturing cycle.	Use of croplands to produce items, not cost-competitive compared to conventional plastics
**Use**	No toxic, no release of chemicals into food if used as packaging	Often characterized by thermal instability, brittleness, low melt strength, high water vapor and oxygen permeability; when hydrophilic polymers are used, they possess low water vapor barrier and vulnerability to degradation.
**Disposal**	Biodegradable; broken down by naturally occurring bacteria; do not persist for many years in the environment.	Controlled fate in order to kickstart the expected biodegradation process; a specific disposal procedure must be followed to avoid they fragment into microplastics which accumulate in the environment.

## Data Availability

Data sharing not applicable.

## Appendix A

**Table A1:** Fruits and vegetables wastes used in the last 10 years as raw material for the production of bioplastics. A full description of the methods used for the production of bioplastics (Technology and pre-treatment of the waste, processing), as well as the main physical and mechanical properties of the obtained materials are provided.

Food Waste Source	Technology and Pre-Treatment	Processing	Bioplastic Type	Bioplastic Mechanical and Physical Properties	Reference
Apple pomace	Apple pomace, either washed with water or not, was powdered. A mixture was prepared containing 2% (*w*/*v*) of powder and 7% glycerol (*w/w* of apple pomace powder) and dissolved in 1% (*w*/*v*) of citric acid solution under heating (70 °C) and constant magnetic stirring at 560 rpm. Then, the mixture was poured onto a non-sticky plate for casting. A mixture without glycerol was also used in order to prepare a biofilm.	Casting	Pectin based biofilm	Tensile strength (MPa): 3.27–16.49 Elongation at break: 10.77–55.41%	Gustaffson et al. [88]
	Apple pomace, either washed with water or not, was powdered. A mixture was prepared containing glycerol and apple pomace powder (70:30) was prepared. 40 g of the mixture was placed into a mold. A pressure of 8 MPa was applied for 20 min at 100 °C.	Molding	Pectin based 3D biomaterial	Tensile strength (MPa): 3.02–5.79 Young’s Modulus (MPa): 367.1–633.4 Elongation at break: 0.93–1.56%	Gustaffson et al. [88]
Banana peels and pseudostems	Banana peels were boiled in water for about 30 min; then, they were left to dry and squashed to obtain a paste. 25 g of banana paste were placed in a beaker with 3 mL of HCl (0.1 N) and 2 mL of glycerol and stirred. Subsequently, NaOH (0.1 N) was added in order to neutralize the pH up to 7.	Casting	Cellulose based biofilm	Weight loss after 13 days: 0.04 g Difference of weight after swelling test (g): 0.01–0.10	Yaradoddi et al. [89]
	Banana peels were washed, sliced, and blended. Then, they were grounded and ground sample was heated at 150 °C for 2 h at 30 psi. The obtained paste was hydrolyzed (100 mL/50 g sample) by (HCl, 99%). The sample was filtrated and washed with water. 1000 g of sample were mixed with chlorinated paraffin liquid plasticizers (1:8 of sample), 5% acetic acid (5 mL/100 g sample), 5 mL/100 g of polyvinylchloride, cellulose (25%) and 25% starch powder, 5% toluene Phthalates ester and 10%water. Then, 10 mL/100 g of PVC and glycerine were added. The mixture was heated at 150 °C in the oven for 30 min at 30 psi pressure.	Casting	Biocomposite	Tensile strength (MPa/kg·m^3^): 120.0 Tensile modulus (GPa): 1.1 Water absorption: 0.03%	Sharif Hossain et al. [139]
	Banana peels were immersed in a Na_2_S_2_O_5_ solution (1% *w*/*v*) for 24 h, oven-dried at 60 °C and milled. 100 g of milled peels were washed three times with ethanol, then washed in 200 mL of acetone. The pectin was extracted with a citric acid solution at pH 2.0 at 87 °C for 160 min and then centrifuged. The supernatant pH was adjusted to 3.5 with KOH, added with ethanol, stirred for 30 min and left to precipitate at 4 °C. The pellet was washed with ethanol 70%, and dried at room temperature. Then, it was stirred and its pH adjusted to 7, and it was again dried and milled. For the extraction of cellulose nanocrystals (CNCs), the alcohol insoluble residue of banana peels was suspended in a mixture of 93 wt% acetic acid and 0.3 wt% HCl in distilled water. Subsequently, the pulp was rinsed and washed in more steps and an acid hydrolysis was conducted with a 30 v% H_2_SO_4_ solution at 45 °C, for 150 min. After centrifugation-dilution-sonication cycles, the CNC suspension was dialyzed against deionized water. CNCs (at different wt%), 4.5 g of pectin, 1.35 g glycerol, citric acid, and distilled water were mixed to form biofilms.	Casting	Pectin based biofilm	Tensile strength (MPa) without citric acid: 7.36 ± 1.15 Tensile strength (MPa) with citric acid: 7.92 ± 1.21 Elongation at break (%)without citric acid: 4.69 ± 0.84 Elongation at break (%)with citric acid: 4.26 ± 0.77 Elastic modulus (MPa)without citric acid: 1586 ± 487 Elastic modulus (MPa)with citric acid: 1714 ± 452 Water vapor permeability(g·mm·kPa^−1^·h^−1^·m^−2^) without citric acid: 3.31 ± 0.36 Water vapor permeability(g·mm·kPa^−1^·h^−1^·m^−2^) with citric acid: 3.10 ± 0.33	Oliveira et al. [75]
	300 g of banana peels were dipped in acetic acid solution and then placed into a beaker containing 800 mL water and boiled for 30 min. The water was decanted off and the peels were left to dry. The banana peels were pureed. To 25 mL of the paste, 3 mL of 0.5 M HCl and 2 mL of 15% glycerol solution were added. The mixture was stirred, 3 mL of 1% corn starch and 3 mL of 0.5 M NaOH were added to the mixture and stirred again.	Casting	Starch based biofilm	Tensile strength (N/m^2^): 12.22–34.72 Water uptake: 60.65–108.98%	Sultan et al. [90]
	Banana pseudostem was sliced, dryer at 50 °C for 5 h and milled. Then, 10 g of pseudo-stem flour were soaked in 300 mL of 5% KOH, centrifuged and bleached with 200 mL of 1% NaClO_2_ at pH 5 for 1 h in 70 °C. 9 g of the bleached pseudo-stem were mixed with TEMPO solution and 22.5 mL of 12% NaClO. At the end of the reaction, the mixture was homogenized and sonicated. Water containing 0.7% solid nanocellulose was mixed with glycerol, nano-clay or graphene oxide in different proportions.	Casting	Biocomposite	Tensile strength (MPa): ~5–~39 Elongation at break (%): ~1–~9 Oxygen permeability (mL/m^2^*day*Pa): 2 × 10^−6^–3.5 × 10^−6^ Water vapor permeability (g/m^2^*day*Pa): ~0.007–~0.023 Contact angle at 20 s (°): 21.89–75.03	Faradilla et al. [91]
	Banana peels were boiled for 60 min and then left to dry and blended. In order to obtain a chemical-based material, 100 g of banana paste were mixed with 12 mL of HCl, 8 mL of glycerol and 12 mL of NaOH. The mixture was stirred for 5 min. Alternatively, a natural-based material was obtained by mixing 40 g of banana peels paste with, 1 g of sage, 12 g of glycerol, 12 g of potato starch, 12 g of corn starch and 38 g of water. The mixture was dried using the oven at a temperature of 120 °C for 3–4 h.	Casting	Biocomposite	Tensile strength for chemical-based material (KPa): 228 Tensile strength for natural-based material (KPa): 150 Young’s Modulus for chemical-based material (MPa): 1.53 Young’s Modulus for natural-based material (MPa): 1.88 Elongation at break for chemical-based material: 18.77% Elongation at break for natural-based material: 13.97%	Azieyanti et al. [138]
	Banana peels were boiled in water for about 30 min. Water was decanted and the peels were left to dry and then they were squashed to obtain a uniform paste. 25 g of banana paste were mixed with 3 mL of (0.1 N) HCl, 2 mL of glycerol and then 3 mL of 0.1 N NaOH to neutralize the pH up to 7.	Casting	Starch based biofilm	Not reported	Rizwana Beevi et al. [113]
Carrots waste	Carrots waste powder was dispersed in a 5% (*w/w*) HCl water solution at a concentration of 50 mg/mL under vigorous stirring at 40 °C. After 12 h, the viscous dispersion was dialyzed using a 3500 MWCO membrane against MilliQ water for 72 h and then cast on a petri dish.	Casting	Cellulose based biofilm	Young’s Modulus (MPa): ~1300 Elongation at break: 6% Ultimate strength (MPa): ~37 Water contact angle: >100° Oxygen permeability (cm^3^μm/m^2^ day kPa): 96 × 10^4^	Perotto et al. [111]
Cassava peels	100 g of cassava peels were washed and soaked in sodium metabisulphyte 1%. Then, they were crushed with 100 mL of water. Slurry resulted was extracted with water (ratio of 1:1) two times. The extracts were precipitated for 3 h. The precipitate was dried; then 5 g were mixed with glacial acetic acid 1%, chitosan (20–50%), glycerol (30%), liquid smoke (0–2 mL) and stirred at 70 °C.	Casting	Biocomposite	Tensile strength (MPa): 35.28–96.04 Elongation at break (%): 14.87–52.27 Water resistance (%): 22.68–78.40	Fathanah et al. [117]
	5.0 g of dried cassava peels waste were mixed with 1.5 mL of glycerol, 0.5 mL of kaffir lime essential oil and 0.7 g of citric acid. The mixture was stirred for 45 min, and heated to 80 °C. Two other samples were prepared without essential oil and without citric acid, respectively.	Casting	Starch based biofilm	Tensile strength (N/cm): 0.3–2.5	Masruri et al. [115]
	Cassava peels were mashed into a pulp. Then, 10 g were extracted with 50 mL of water. The extract was washed with water. Then, the juice precipitated and dried under direct sunlight to form a flour or starch. 3 g of starch were mixed with glycerol (25% wt)) and chitosan (2 and 3% wt). The mixture was heated at a 80–90 °C and stirred.	Casting	Biocomposite	Tensile strength (Kgf/cm^2^): 4.16–27.41 Elongation at break (%): 30.37–94.25	Dasumiati et al. [116]
Cauliflower waste	Cauliflower waste powder was dispersed in a 5% (*w/w*) HCl water solution at a concentration of 50 mg/mL under vigorous stirring at 40 °C. After 12 h, the viscous dispersion was dialyzed using a 3500 MWCO membrane against MilliQ water for 72 h and then cast on a petri dish.	Casting	Cellulose based biofilm	Young’s Modulus (MPa): ~500 Elongation at break: 4% Ultimate strength (MPa): ~7 Water contact angle: ~80°	Perotto et al. [111]
Citrus waste	Citrus peel derived pectin powder was mixed with glycerol (30% (*w/w*)) for 2 min to get a uniform dough which was then formed into ball shape (total weight of 2.5 g). The obtained blend was placed in molding press. The compression molding process was performed for 10 min under operation conditions of 1.33 MPa and 120 °C. Pectin-based biomass films were also produced by incorporation of lyophilized and milled fungal biomass. Biomass concentrations varied in the range of 0–35% of the total mixture. Glycerol content was kept at 30%.	Molding	Pectin based biofilm	Tensile strength (MPa) without fungal biomass: 15.7 ± 0.5 Elongation at break without fungal biomass (%): 5.5 ± 1.7 Young’s Modulus (MPa) without fungal biomass: 298 ± 58 Tensile strength (MPa) with fungal biomass: 5.2–19.3 Elongation at break with fungal biomass (%): 1.4–4.5 Young’s Modulus (MPa) with fungal biomass: 187–1350 Water Vapor Permeability Coefficient ((kg·s^−1^·m^−1^·Pa^−1^) × 10^13^) without fungal biomass: 7 Water Vapor Permeability Coefficient ((kg·s^−1^·m^−1^·Pa^−1^) × 10^13^) with fungal biomass: ~2–4	Gurram et al. [102]
Cocoa pod husks	Cocoa pod husks were washed to remove residual sugars and alcohols and then dried in an oven at 40 °C overnight. Solutions of 3% by weight of solids in TFA were prepared in glass vials. Vials were sealed with Parafilm and were placed in a benchtop lab shaker for 29 days. The obtained solution was centrifuged in order to remove any residuals.	Casting	Cellulose based biofilm	Tensile stress at break (MPa): 70 Water adsorption %: <10 (at a relative humidity <50%) Water adsorption %: >10 (at a relative humidity >80%) Initial decomposition temperature under 44% of relative humidity (°C): ~200	Bayer et al. [37]
	100 g of milled cocoa by-products were placed in 400 g of diethyl ether under stirring at room temperature for 24 h. After evaporation, the supernatant gave a first residue. In addition, the solid residue was separated by filtration and, subsequently, underwent the second extraction with 400 g of ethanol under stirring at room temperature for 24 h. Once again, the supernatant was evaporated and the second residue was collected. The third residue was used as obtained after filtration.	Extrusion	Biocomposite	Oxygen permeability [cc·mm/(m^2^·bar·24 h)]: 844–1104 for PLA and 982-5784 for PP Wettability (contact angle): 88 ± 2°	Battegazzore et al. [76]
	Cocoa shell waste (CSW) was grinded. The micronized CSW powder was added to 10 mL of heptane and stirred for 2 min. Then, the silicone (Elastosil E43) was added to the CSW dispersion and vigorously stirred for further 5 min to obtain a homogenous blend.	Casting	Biocomposite	Tensile strain at break %: 15–250 Young Modulus (MPa): 1.96 ± 0.13–10.67 ± 0.19 Water vapor permeability (g (m d Pa)^−1^): 1.51·10^−5^–14.93·10^−5^ BOD saturation level (mg O_2_/L): 36.1–38.2	Tran et al. [135]
Hazelnut skin	50 g of hazelnut skin were placed in 400 g of diethyl ether under stirring at room temperature for 24 h. After evaporation, the supernatant gave a first residue. In addition, the solid residue was separated by filtration and, subsequently, underwent the second extraction with 400 g of ethanol under stirring at room temperature for 24 h. Once again, the supernatant was evaporated and the second residue was collected. The third residue was used as obtained after filtration.	Melt-blending extrusion	Biocomposite	Oxygen permeability [cc·mm/(m^2^·bar·24 h)]: 11.7–1875 for PLA and 122-338 for PP Wettability (contact angle): 80 ± 2°	Battegazzore et al. [76]
Hemp fibers	Dried hemp fibers were aggregated with tomato fibers in different percentages (100% tomato fibers, 90% tomato fibers, 70% tomato fibers). 50.0 g of the aggregated were soaked in 100 mL of a 2% (*w*/*v*) sodium alginate water solution.	Molding	Biocomposite	Young Modulus (MPa): 62.51–97.08 Tensile stress at break (MPa): 0.46–1.20 Maximum load (N) *: 8.7–14.8 Displacement (mm) *: 3.22–4.75 Water up-take percentage W_st_%: 128–186 Time for complete biodegradation: 16 days after the transplanting	Schettini et al. [105]
Jackfruit seeds	Jackfruit seeds were removed from the skin of the arrows and then washed and powdered. The obtained powder was mixed with sorbitol (from 0 to 6 mL) and poly vinyl alcohol (from 0 to 3 g).	Not specified	Not specified	Tensile strength (MPa): 0–~2.2 Elongation at break: 0–7%	Lestari et al. [93]
Mangosteen peels	Mangosteen peels were sun dried for about 48 h at room temperature. Then, they were grinded and sieved. To prepare cellulose fibers, about 50 g of the Mangosteen peels powder were treated with 700 mL of 0.1 M NaOH, under heating and stirring. Then the insoluble pulp was bleached with 500 mL of NaOCl buffered to a pH 5 and washed with distilled water. The cellulose fibers were air dried. Then 10 g of fibers were hydrolyzed in 100 mL of 95% H_2_SO_4_ at 500 °C, then diluted with distilled water, centrifuged and sonicated. The resulting suspension cellulose nanocrystals (CNCs) were dried in a freeze drier at 3 °C. CNCs were mixed in different wt% (0–19%) with 10 g of cassava starch, 60 mL of distilled water, 5 mL of vinegar and 7 mL of glycerol. The mixture was stirred at 105 °C up to 200 °C.	Casting	Biocomposite	Tensile strength (MPa): ~1.3–~2 Young’s Modulus (GPa): ~15–~26 Elongation at break (%): ~15–~23	Muhammad et al. [103]
Miscanthus	Chopped 2 mm miscanthus were dried at 80 °C for at least 24 h. They were mixed with oat hull and blends of PBS/PBAT (80/20) in the presence of peroxide (0.02 phr) with feeder at 100 rpm and 180 °C.	Extrusion	Biocomposite	Tensile strength with 20% Mischatus fiber (MPa): 1.0 Tensile strength with 40% Mischatus fiber (MPa): 1.0 Young’s Modulus with 20% Mischatus fiber (MPa): 64 Young’s Modulus with 40% Mischatus fiber (MPa): 404 Water absorption with 20% Mischatus fiber: 1–3% Water absorption with 40% Mischatus fibre: 1–7%	Wu et al. [133]
Oat Hull	Chopped 2 mm oat hull were dried at 80 °C for at least 24 h. They were mixed with miscanthus and blends of PBS/PBAT (80/20) in the presence of peroxide (0.02 phr) with feeder at 100 rpm and 180 °C.	Extrusion	Biocomposite	Tensile strength with 20% oat hull (MPa): 0.4 Tensile strength with 40% oat hull (MPa): 0.29 Young’s Modulus with 20% oat hull (MPa): 54 Young’s Modulus with 40% oat hull (MPa): 257 Water absorption with 20% oat hull: 1–5% Water absorption with 40% oat hull: 2–9%	Wu et al. [133]
Orange peels	Dried orange peels were washed with a HCl (0.03 N) at 60 °C and agitated for 30 min. The residue was hydrolyzed with HCl (0.04 N) at 90 °C for 20 min. Then, pectin extraction was performed with hot water at 90 °C and agitated for 30 min. The obtained pectin jelly was mixed with corn starch, layered silicates, glycerol and water; the mixture was left overnight in an oven at 70 °C.	Extrusion followed by casting	Biocomposite	Equilibrium recoverable compliance (m^2^/N): 3.71 × 10^−9^ Water vapor transmission rate (g/m^2^h): 9.87 Oxygen gas transmission rate (mL/m^2^day): 1366 ± 194	Cokajgil et al. [100]
	Orange waste (OW) was washed with water then dried for 16 h at 40 °C and milled to a fine powder. A mixture of 2% (*w*/*v*) of OW powder was prepared in 1% (*w*/*v*) citric acid solution under constant magnetic stirring at 70 °C. The acid solution also contained 7% (*w/w*) glycerol and 1 drop of organic antifoam/100 mL solution. The suspension was sieved before it was poured onto PTFE plates and dried at 40 °C.	Casting	Pectin and cellulose based biofilm	Tensile strengths (MPa): 28–36 Time for 90% degradation: 15 days	Batori et al. [92]
Palm empty fruit bunch	Palm empty fruit bunch was dried, powdered and cooked for 8 h at 80 °C. 10% NaOH solution was added and the mixture was autoclaved for 15 min at 121 °C. The obtained mixture was added with 10% sodium hypochlorite processed using ultrafine grinder in wet milling method. 2% cellulose was mixed with 2 L of water and passed through the grinder for up to 30 cycles until a nanocellulose gel was formed. 5% of the produced nanocellulose was introduced in the Enviplast^®^ formula.	Extrusion	Biocomposite	Tensile strength (kgf/cm^2^): 191.30 Elongation at break (%): 197.12 Water Vapour Transmission Rate (g/m^2^/24 h): 299.42	Iriani et al. [140]
Parsley stems	Parsley stems were washed to remove residual sugars and alcohols and then dried in an oven at 40 °C overnight. Solutions of 3% by weight of solids in TFA were prepared in glass vials. Vials were sealed with Parafilm and were placed in a benchtop lab shaker for 29 days. The obtained solution was centrifuged in order to remove any residuals.	Casting	Cellulose based biofilm	Tensile stress at break (MPa): 5 Water adsorption %: <10 (at a relative humidity <50%) Water adsorption %: >10 (at a relative humidity >80%) Initial decomposition temperature under 44% of relative humidity (°C): ~200	Bayer et al. [37]
	Parsley stems powder was dispersed in a 5% (*w/w*) HCl water solution at a concentration of 50 mg/mL under vigorous stirring at 40 °C. After 12 h, the viscous dispersion was dialyzed using a 3500 MWCO membrane against MilliQ water for 72 h and then cast on a petri dish.	Casting	Cellulose based biofilm	Young’s Modulus (MPa): ~200 Elongation at break: 10% Ultimate strength (MPa): ~7 Water contact angle: ~60	Perotto et al. [111]
Passion fruit peels	Passion fruits peels were dried and milled to fine flour. A mixture of corn and cassava starches, 45 and 55%, respectively, was placed in a homogenizer and blended for 10 min with the passion fruit peel. 2000 g of the mix was processed by the extruder. For the plasticizer solutions (1 L for each treatment), different amounts of glycerol and water were prepared (60, 64, 70, 76, and 80% glycerol content). The extrudates were cut into 5-g pieces, placed between Teflon sheets, compressed, and molded at 5 ton and 90 °C for 30 s.	Extrusion followed by molding	Biocomposite	Tensile strength (MPa): 1.6–9.0 Elongation at break: 24.7–54.5% Young’s Modulus (MPa): 2.4–29.9 Water vapor permeability (g·mm/m^2^·h·kPa): 0.256–0.436 Water solubility index: 50.4–68.3% Contact angle (°): 5.3–72.2	Moro et al. [132]
Peanut hulls	Peanut hulls, were stored at 4 °C, and then reduce to powder. The mixture including 17 g of peanut hulls mashed to a size of around 100 microns was blended minutes, gradually adding potato flour from skins (30 g), whole milk (48 mL) and glycerol (5 mL). The mixed ingredients formed a compound that was cooked in a fan-assisted oven at 180 °C for 13 min.	Casting	Biocomposite	Weight loss %: 6.5 ± 0.5	Troiano et al. [134]
Pine flower waste	Pine flowers were soaked water at 100 °C for 2 h, washed and dried for 24 h at 50 °C. Then, they were grounded and soaked in hot water for 4 h and roasted for 24 h at 60 °C. For cellulose isolation, 50 g of grounded pine flower were mixed with 500 mL of 6% NaOH at 70 °C for 4 h. After serial washing and filtration steps, 5 g of obtained cellulose were mixed with 10%, 30%, and 60% citric acid in 100 mL respectively and ultrasonicated. Then, a polyvinyl alcohol (PVA) solution was prepared by mixing 4% PVA, 25% glycerol, and 71% distilled water (*w/w*), and a starch solution by mixing 3% starch, 12% glycerol and 85% distilled water (*w/w*). Both solutions were mixed together (PVA: starch ratio of 80:20 (*w/w*)) and finally 1% nanocellulose, 10% turmeric extract and 10% natural dragon fruit extract were added.	Casting	Biocomposite	Not reported	Nasihin et al. [137]
Pineapple leaf	Pineapple leaf were scaffed and then the fibers were cut into small sizes and grinded. The grinded fibers were then sieved to get the highest amount of fiber length available. Then they were placed in oven for 24 h and subsequently mixed with tapioca-based bioplastic resin (70–90% *w/w*). The mixture was first extruded into pellet at 160 °C. The pellets were then placed in the mold and hot pressed at 160 °C for 5 min at 8 MPa, and then cold pressed at room temperature for 5 min at 8 MPa.	Extrusion followed by molding	Biocomposite	Tensile Modulus (GPa): 1.029–1.145 Tensile strain (mm/mm): 0.007–0.011	Mathivanan et al. [130]
Potato peels	Potato peels were granulated and centrifuged at 15000 rpm for 20 min. The supernatant was filtered and the starch was obtained. 13.5 g of dried starch was extracted from 330 g wet potato peels. After filtration, starch was dried at 50 °C for 2 h. 13.5 g of starch were mixed with 135 mL of tap water, 16.2 mL of vinegar, and 10.8 mL of glycerin. The mixture was heated (100 °C) and kept waiting at that temperature for 20 min.	Casting	Starch based biofilm	Water absorption: 48.46% within two hours and 83.57% within 24 h Time for complete biodegradation: 28 days in moist soil.	Arikan et al. [114]
	Potato peels were boiled with water and then the starch was extracted from the water by filtration. The starch was mixed with glycerine, vinegar and water in different ratio, at 105 °C.	Casting	Starch based biofilm	Resistence to compressive stress (MPa): 0.5–1.1	Samer et al. [118]
Pumpkin peels	1 g of dry pumpkin peel residue was suspended in 50 mL of 2 wt% NaOH solution and stirred for 4 h at 100 °C. Then 80 mL of 0.5 M NaOH containing 2% (*v*/*v*) of H_2_O_2_ per each gram of material were added. The solution was stirred during 1 h. Then, 20 mL of 2 M NaOH solution was added and the suspension stirred during 5 h at 55 °C. Subsequently, the residue was washed with distilled water until achieve neutral pH. Finally, the residue was filtered and dried. 12 mL of [(BMIM)Cl] was used per each 0.25 g of material to dissolute cellulose. The isolated cellulose was acetylated and 0.02 g were added to a solution of PLA (0.18 g) in DCM (25 mL). The mixture was stirred for 1 h, at 50 °C and then it was casted.	Casting	Biocomposite	Storage Moduls (GPa): 1.85 at 40 °C	Coto et al. [136]
Radicchio waste	Radicchio waste powder was dispersed in a 5% (*w/w*) HCl water solution at a concentration of 50 mg/mL under vigorous stirring at 40 °C. After 12 h, the viscous dispersion was dialyzed using a 3500 MWCO membrane against MilliQ water for 72 h and then cast on a petri dish.	Casting	Cellulose based biofilm	Young’s Modulus (MPa): ~200 Elongation at break: 5% Ultimate strength (MPa): ~5 Water contact angle: ~80°	Perotto et al. [111]
Rice straw and hulls	Rice hulls were washed to remove residual sugars and alcohols and then dried in an oven at 40 °C overnight. Solutions of 3% by weight of solids in TFA were prepared in glass vials. Vials were sealed with Parafilm and were placed in a benchtop lab shaker for 29 days. The obtained solution was centrifuged in order to remove any residuals.	Casting	Cellulose based biofilm	Tensile stress at break (MPa): 7 Water adsorption %: <10 (at a relative humidity <50%) Water adsorption %: >10 (at a relative humidity >80%) Initial decomposition temperature under 44% of relative humidity (°C): ~225	Bayer et al. [37]
	200 g of dried rice straw samples were placed in a solid-liquid extractor with 1 L of Milli-Q water. Total extraction was performed for approximately 3 h and with 30 cycles and 12 strikes per cycle and the static phase for 10 min. Then, 10 g of the powdered and dried rice straw, previously washed and dried, was mixed with 200 mL of TFA and maintained under magnetic stirring (800 rpm) at room temperature for 3 days and, poured into a low edge crystallizing container maintained under laminar hood.	Casting	Cellulose based biofilm	Tensile strength at break (MPa): 45 (dried dumbbells) Elongation at break (%): 6.1 (dried dumbbells) Tensile strength at break (MPa): 10 (wet dumbbells) Elongation at break (%): 63 (wet dumbbells) Water adsorption %: 40.7–42.6 Time for complete biodegradation: 105 days in soil.	Bilo et al. [110]
	Rice straw was dried, powdered and cooked for 8 h at 80 °C. 10% NaOH solution was added and the mixture was autoclaved for 15 min at 121 °C. The obtained mixture was added with 10% sodium hypochlorite processed using ultrafine grinder in wet milling method. 2% cellulose was mixed with 2 L of water and passed through the grinder for up to 30 cycles until a nanocellulose gel was formed. 5% of the produced nanocellulose was introduced in the Enviplast^®^ formula.	Extrusion	Biocomposite	Tensile strength (kgf/cm^2^): 168.36 Elongation at break (%): 156.36 Water Vapour Transmission Rate (g/m^2^/24 h): 301.06	Iriani et al. [140]
	Rice straw were chopped to prepare the fibres with lengths ranging from 0.5 to 2 mm. The fibers were dehydrated for 24 h within a vacuum oven at 105 °C. Three chosen polymers (PLA, Lignin, PP) were separately compounded with 20% of rice straw fiber.	Extrusion	Biocomposite	Not reported	Dahy et al. [142]
	Rice waste powdered. The powder of rice waste was mixed with chitosan (from 30 to 60%) and glycerol (from 0 to 3 mL), heated at 50–60 °C for 30 min.	Molding	Cellulose based biofilm	Tensile strength (MPa): ~0–60 Elongation at break: 2–4% Water resistance: 0.1–0.6%	Lestari et al. [93]
Sago waste	Sago waste was soaked in hot water at 40 °C for 2 h and air dried. The sago waste was then treated with 2% NaOH aqueous solution at 60 °C for 1 h, filtered and washed. The samples were dried at 40 °C. The obtained fibers were bleached with NaClO_2_/glacial CH_3_COOH mixture at 80 °C. Then, they were washed with distilled water and dried at 60 °C. The cellulose fibers were dissolved in 10 mL of distilled water, stirred, ultrasonicated for 30 min and, subsequently, mixed with a sago starch 4% (*w/w*) solution.	Casting	Biocomposite	Tensile strength (MPa):86.66–123.03 Young’s Modulus (MPa): 1710–2958 Elongation at break (%): 3.85–4.62 Water absorption (%): ~100–~200	Yacob et al. [141]
Soy waste	Soy waste was bleached with a solution of distilled water:sodium hypochlorite (70:30). Then, it was separated from the solvent, rinsed with distilled water and dried in the oven for one hour at 100 °C. Subsequently, it was powdered and 3.0 g were mixed with corn starch (9.5 g), glycerol (5 mL), vinegar (5 mL) and water (60 mL). The mixing process was carried out at 25 °C and 50 rpm for 10 min.	Casting	Biocomposite	Maximum value of force before fracture (N): 6.71 Water absorption (%): 114.17	Muhammad et al. [119]
Spinach steams	Spinach steams were washed to remove residual sugars and alcohols and then dried in an oven at 40 °C overnight. Solutions of 3% by weight of solids in TFA were prepared in glass vials. Vials were sealed with Parafilm and were placed in a benchtop lab shaker for 29 days. The obtained solution was centrifuged in order to remove any residuals.	Casting	Cellulose based biofilm	Tensile stress at break (MPa): 1 Water adsorption %: <10 (at a relative humidity <50%) Water adsorption %: >10 (at a relative humidity >80%) Initial decomposition temperature under 44% of relative humidity (°C): ~130	Bayer et al. [37]
Tea leaves waste	Tea leaves waste were dried under vacuum at 70 °C, grounded, and sifted. Tea waste powder (TW) was dried under vacuum at 70 °C. TW bioplastics were synthesized with 1 g of TW powder in 20 mL of 3% citric acid solution (TW-CA) or only with water (TW-H2O). Carboxymethylcellulose sodium salt at 5% was investigated also as an additive to TW bioplastics (TW-CMC). All samples were magnetically stirred in an oil bath at 60 °C for 12 h, then casted.	Casting	Cellulose based biofilm	Ultimate tensile strength (MPa): 2–~6 Elongation at break: 0.5%–~13% Water contact angles (°): ~40–~120	Liu et al. [112]
Tomato waste	After the extraction of polysaccharides, carotenoids and polyphenols from peels and seeds, the residual dried fibers were combined with hemp fibers in different percentages (0% hemp fibers, 10% hemp fibers, 30% hemp fibers). 50.0 g of the aggregated fibers were soaked in 100 mL of a 2% (*w*/*v*) sodium alginate water solution.	Molding	Biocomposite	Young Modulus (MPa): 62.51–97.08 Tensile stress at break (MPa): 0.46–1.20 Maximum load (N) *: 8.7–14.8 Displacement (mm) *: 3.22–4.75 Water up-take percentage W_st_%:128–186 Time for complete biodegradation: 16 days after the transplanting	Schettini et al. [105]
	Tomato pomace was subjected to alkaline hydrolysis (100 °C for 6 h with a NaOH 0.5 M solution in water) to obtain cutin monomers. The supernatant was discarded and the resulting solution was acidified with HCl 3 M up to final pH 3. 80.0 mg of tomato pomace monomers were blended with tin (II) 2-ethylhexanoate; the mixtures were placed on open carbon-doped Teflon molds and heated in air inside a convention oven.	Melt polycondensation	Aliphatic polyester	Young Modulus (MPa): 14–214 Hardness (MPa): 1.8–26.3 Water contact angle: 81°–109° Water up-take percentage: 2.1–6.1%	Heredia-Guarreiro et al. [124]
